# Supplementary material for: A UMLS-based spell checker for natural language processing in vaccine safety
Source: BMC Med Inform Decis Mak. 2007 Feb 12;7:3. doi: 10.1186/1472-6947-7-3 (PMC1805499; doi:10.1186/1472-6947-7-3)
Supplement: Additional file 13 — RAP application directory. Sets up directory for RAP (RDF application for PHP) source code [file 1472-6947-7-3-S13.gz › rap/doc/tests/test_ResModel.html]

### RDF API for PHP V0.9.1

# Test: Using the ResModel

  
This example is part of the RAP - Rdf API for PHP documentation.

Setting up the model:

//change the RDFAPI\_INCLUDE\_DIR to your local settings  
define("RDFAPI\_INCLUDE\_DIR", "C:/!htdocs/rdfapi-php/api/");   
include(RDFAPI\_INCLUDE\_DIR . "RdfAPI.php");

// Some definitions  
define('VCARD\_NS', 'http://www.w3.org/2001/vcard-rdf/3.0#');  
$personURI = "http://somewhere/JohnSmith";  
$fullName = "John Smith";

// Create an empty Model   
$model = ModelFactory::getResModel(MEMMODEL);

// Create the resources  
$fullNameLiteral = $model->createLiteral($fullName);  
$johnSmith = $model->createResource($personURI);  
$vcard\_FN= $model->createProperty(VCARD\_NS.'FN');  
$vcard\_NICKNAME= $model->createProperty(VCARD\_NS.'NICKNAME');

// Add the property   
$johnSmith->addProperty($vcard\_FN, $fullNameLiteral);

// Retrieve the John Smith vcard resource from the model   
$vCard = $model->createResource($personURI);

// Retrieve the value of the FN property   
$statement = $vCard->getProperty($vcard\_FN);  
$value = $statement->getObject();

// Add two nickname properties to vcard  
$literal1 = $model->createLiteral("Smithy");  
$literal2 = $model->createLiteral("Adman");   
$vCard->addProperty($vcard\_NICKNAME, $literal1);  
$vCard->addProperty($vcard\_NICKNAME, $literal2);

  

// List the nicknames  
echo '<b>Known nicknames for '.$fullNameLiteral->getLabel().':</b><BR>';  
foreach ($vCard->listProperties($vcard\_NICKNAME) as $currentResource)   
{   
    echo $currentResource->getLabelObject().'<BR>';  
};

**Known nicknames for John Smith:**  
Smithy  
Adman

echo '<BR><b>Iterate over all subjects which having FN property:</b><br>';   
// Iterate over all subjects which having FN property  
$iter = $model->listSubjectsWithProperty($vcard\_FN);   
for ($iter->rewind(); $iter->valid(); $iter->next())  
{  
    $currentResource=$iter->current();  
    echo $currentResource->getLabel().'<BR>';  
};

**Iterate over all subjects which having FN property:**  
http://somewhere/JohnSmith

Create a Bag:

// Create a bag   
$bag\_smiths = $model->createBag();

$beckySmith = $model->createResource('http://somewhere/BeckySmith');  
$beckySmithFN = $model->createLiteral('Becky Smith');  
$beckySmith->addProperty($vcard\_FN,$beckySmithFN );

// Add persons to bag  
$bag\_smiths->add($beckySmith);  
$bag\_smiths->add($johnSmith);

// Print out the full names of the members of the bag  
echo '<BR><BR><b>Print out the full names of the members of the bag:</b></BR>';   
foreach ($bag\_smiths->getMembers() as $resResource)  
{  
    // Retrieve the value of the FN property   
    $statement = $resResource->getProperty($vcard\_FN);  
    echo $statement->getLabelObject().'<BR>';   
};

**Print out the full names of the members of the bag:**
Becky Smith  
John Smith

echo '<BR><BR>All Statements as HTML table';  
$model->writeAsHTMLTable();

All Statements as HTML table

|  |  |  |  |
| --- | --- | --- | --- |
| **Base URI:** | | | **Size:** 7 |
| **No.** | **Subject** | **Predicate** | **Object** |
| 1. | Resource: http://somewhere/JohnSmith | Resource: http://www.w3.org/2001/vcard-rdf/3.0#FN | Literal: John Smith |
| 2. | Resource: http://somewhere/JohnSmith | Resource: http://www.w3.org/2001/vcard-rdf/3.0#NICKNAME | Literal: Smithy |
| 3. | Resource: http://somewhere/JohnSmith | Resource: http://www.w3.org/2001/vcard-rdf/3.0#NICKNAME | Literal: Adman |
| 4. | Resource: http://somewhere/BeckySmith | Resource: http://www.w3.org/2001/vcard-rdf/3.0#FN | Literal: Becky Smith |
| 5. | Blank Node: bNode1 | RDF Node: http://www.w3.org/1999/02/22-rdf-syntax-ns#type | RDF Node: http://www.w3.org/1999/02/22-rdf-syntax-ns#Bag |
| 6. | Blank Node: bNode1 | RDF Node: http://www.w3.org/1999/02/22-rdf-syntax-ns#\_1 | Resource: http://somewhere/BeckySmith |
| 7. | Blank Node: bNode1 | RDF Node: http://www.w3.org/1999/02/22-rdf-syntax-ns#\_2 | Resource: http://somewhere/JohnSmith |
